# A Rare Entity–Percutaneous Lead Extraction in a Very Late Onset Pacemaker Endocarditis: Case Report and Review of Literature

**DOI:** 10.3390/diagnostics11010096

**Published:** 2021-01-09

**Authors:** Andreea Maria Ursaru, Cristian Mihai Haba, Ștefan Eduard Popescu, Daniela Crișu, Antoniu Octavian Petriș, Nicolae Dan Tesloianu

**Affiliations:** 1 Department of Cardiology, Emergency Clinical Hospital “Sf. Spiridon”, 700111 Iași, Romania; cristi.haba@gmail.com (C.M.H.); daniellacrisu@yahoo.com (D.C.); antoniu.petris@yahoo.ro (A.O.P.); dan_tesloianu@yahoo.com (N.D.T.); 2 Department of Cardiology, “Grigore. T. Popa” University of Medicine and Pharmacy, 700115 Iași, Romania

**Keywords:** cardiac device, endocarditis, infection, late lead extraction, pacemaker lead endocarditis, late lead-related infective endocarditis

## Abstract

The number of infections related to cardiac implantable electronic devices (CIEDs) has increased as the number of devices implanted around the world has grown exponentially in recent years. CIED complications can sometimes be difficult to diagnose and manage, as in the case of lead-related infective endocarditis. We present the case of a 48-year-old male diagnosed with Staphylococcus aureus device-related infective endocarditis, 12 years after the implant of a single chamber pacemaker. A recent history of the patient includes two urinary catheterizations due to obstructive uropathy in the context of a prostatic adenoma, 2 months previously, both without antibiotic prophylaxis; no other possible entry sites were found and no history of other invasive procedures. After initiation of antibiotic therapy according to antibiotic susceptibility testing, we decided to remove the right ventricular passive fixation lead along with the vegetation and pacemaker generator; because of severe lead adhesions in the costoclavicular region, and especially in the right ventricle, we needed mechanical sheaths to remove the abundant fibrous tissue that encompassed the lead. After a difficult, but successful, lead extraction along with a large vegetation and 6 weeks’ antibiotic therapy, the clinical and biological evolution was favorable, without reappearance of symptoms. While very late lead endocarditis is a rarity, late lead-related infective endocarditis (more than 12 months elapsed since implant) is not an exception; this is why we find that endocarditis prophylaxis should be reconsidered in certain patient categories, our patient being proof that procedures with neglectable endocarditis risk according to the guidelines can lead to bacterial endocarditis.

## 1. Introduction

Infection of cardiac implantable electronic devices (CIEDs) is a severe pathology associated with high mortality. The majority of patients necessitating these types of devices are rather elderly, with multiple comorbidities. Put together, the individual characteristics and the increasing number of patients requiring CIEDs lead to increased CIED-related infections [1].

As lead-related infective endocarditis is not an uncommon pathology anymore, an early diagnosis is essential, since multiple complications may appear with the ongoing infectious process: pulmonary embolism, infiltrates, abscess, cavitation, aneurysms, systemic embolism and infarcts (paradoxical embolus via patent foramen ovale or intracardiac shunt), conduction system disturbances (most often atrioventricular block), and arrhythmias, septic shock, multiorgan failure, or local involvement such as tricuspid valve insufficiency or stenosis, valvular destruction, perforation, or abscess formation [2,3]. The removal of the entire pacing system should be performed rather than attempting prolonged antibiotic therapy alone, both for systemic infection and for infection of the pacemaker pocket, since the mortality rates for infected devices decreases from 31% to 66% when the device is not explanted [4] to under 18% when a combined management with entire system removal and antimicrobial therapy is adopted [5]. The relapse rate among patients without complete removal of the infected material is very high. Percutaneous lead extraction is the preferred initial approach. Late lead extraction in CIED endocarditis can be challenging especially due to fibrotic adhesions that develop over time and increase the risk of complications: tricuspid valve injury, cardiac tamponade, subclavian vein laceration, hemothorax, massive hemorrhage, lead fracture, or septic embolic phenomena [6,7]. Procedures done in centers with experience are associated with significantly lower major all-cause in-hospital complications and death, which is why this type of high-risk patient should be referred to high-volume hospitals. Surgical lead extraction is reserved for large vegetations or failed attempts at percutaneous removal [8].

## 2. Case Report 

We present the case of a 48-year-old male who was admitted to a local county hospital for fever, chills, and extreme fatigability; a routine blood test revealed inflammatory syndrome and severe anemia and after 24 h, the blood cultures became positive for methicillin-susceptible Staphylococcus aureus. The recent history of the patient described a similar episode, two weeks previously, with fever, headache, and accentuated fatigability alleviated under empirical antibiotic therapy with amoxicillin/clavulanic acid 2 g/day for 5 days. Two months previously, the patient presented two consecutive episodes of urinary obstruction within 10 days; each time he underwent urinary catheterization without antibiotic prophylaxis for infective endocarditis, since, according to guidelines, he was framed as a negligible-risk patient, and the patient’s history included a single chamber pacemaker implant for intermittent complete atrioventricular (AV) block, 12 years previously (2008). Considering the patient’s history and the positive blood cultures, a transthoracic echocardiography was performed, which revealed a 21/10 mm pedunculated hypoechogenic mobile mass attached to the tricuspid valve. After establishing the diagnosis of right-sided bacterial endocarditis, the antibiotic treatment was adapted, with initiation of oxacillin 12 g/day, rifampicin 900 mg/day, and gentamicin 3 mg/kg/24 h. The patient was then redirected from the local county hospital to our center for further investigations and treatment. 

At admission to our clinic, the patient was in poor condition, feverish (38.8 °C), blood pressure of 110/70 mmHg, heart rate of 82 beats per minute (bpm), regular, with pale skin and mucous membranes and extreme fatigability. Generator site was without inflammatory signs, and normal scar formation. Biological, severe normochromic normocytic anemia, (hemoglobin 6.6 g/dL) with normal values of leucocytes (8.34 × 10^3^/μL), but increased inflammatory markers (C-reactive protein 4.62 mg/dL, ferritin 1066 ng/mL, fibrinogen 800 mg/dL) and elevated serum creatinine (2.63 mg/dL). 

**Figure 1:**
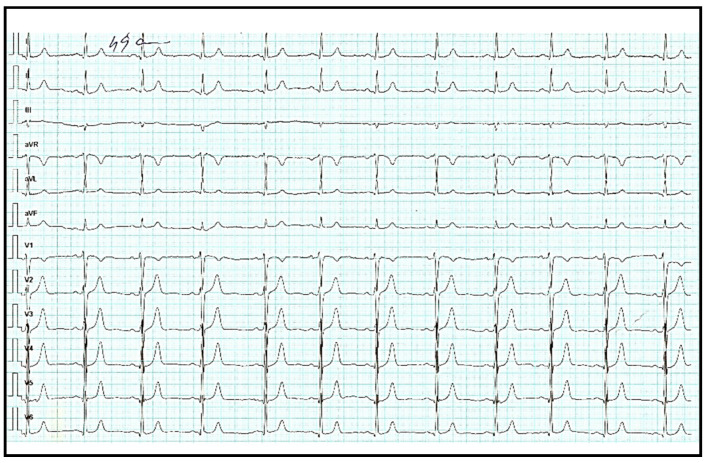
Electrocardiogram revealed sinus rhythm, heart rate 75 beats per minute, intermediate QRS axis, normal morphology.

The electrocardiogram (ECG) showed sinus rhythm, heart rate 75 bpm, intermediate QRS axis, normal morphology (Figure 1).

**Figure 2:**
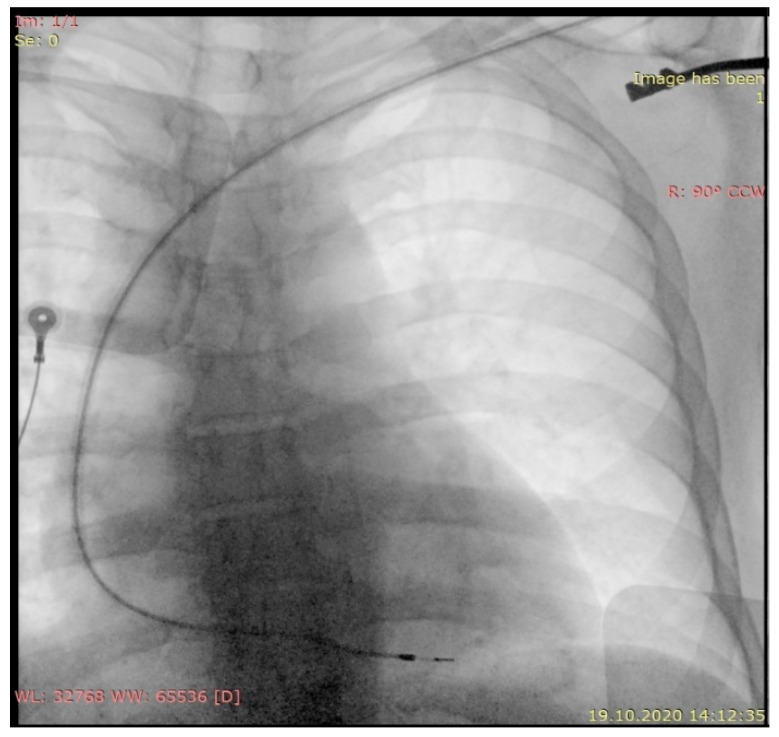
Antero-posterior chest X-ray: passive fixation lead on the topography of the right ventricle.

Pacemaker interrogation pointed out the end of life (EOL) of the battery and the device ID card also mentioned EOL in the last year. Twenty-four-hour Holter ECG monitoring revealed the presence of sinus rhythm without sinus pauses or episodes of atrioventricular block throughout the entire period. Chest X-ray showed a passive fixation pacemaker lead at the apex of the right ventricle, no lead fracture (Figure 2). 

**Figure 3:**
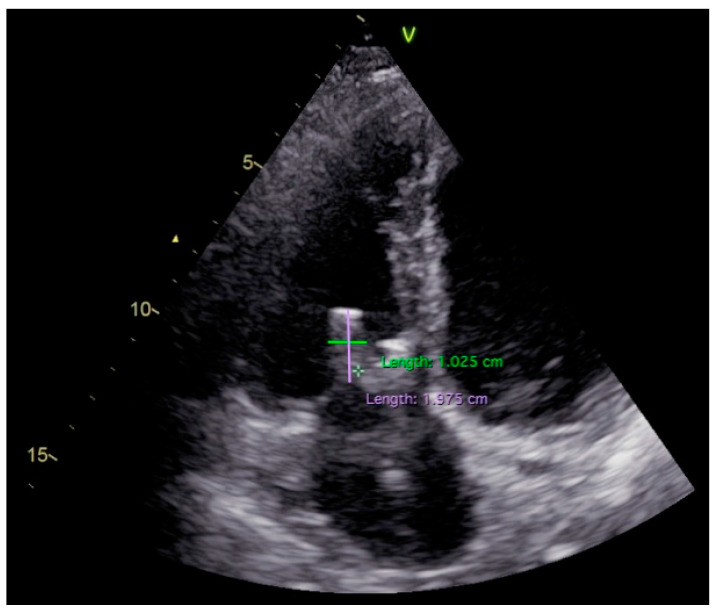
Transthoracic two-dimensional echocardiography apical four-chamber view: large hypoechogenic hyper-pedunculated mobile mass (19.5/10 mm) at the level of tricuspid valve.

**Figure 4:**
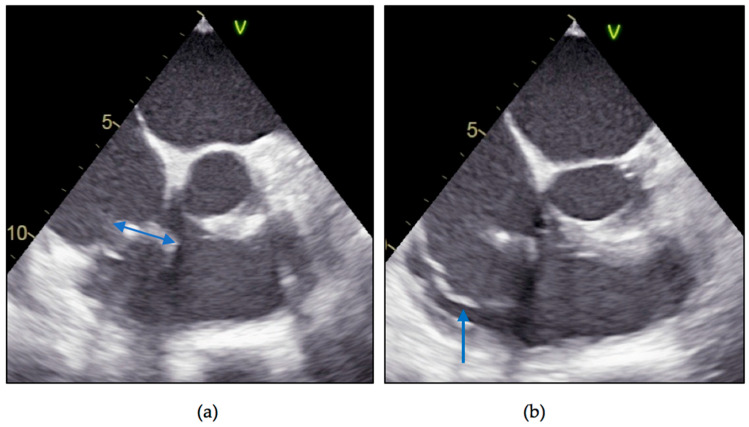
Transesophageal two-dimensional echocardiography: (**a**) pedunculated mobile mass (blue, double-headed arrow) attached to pacing lead (19/5 mm); (**b**) posterior leaflet of the tricuspid valve with hypermobility and rupture of chordae (blue arrow).

Transthoracic echocardiography described a similar appearance to the initial echo exam, with a mobile hypoechogenic mass at the level of the posterior leaflet of the tricuspid valve/pacemaker lead, with a small decrease in dimensions of 19.5/10 mm (Figure 3; Video 1, Supplementary File) and mild tricuspid regurgitation; dilated inferior vena, with inspiratory collapse, preserved left and right ventricular systolic function. The transthoracic approach could not establish the exact location or the singularity of the vegetation. As indicated, we proceeded with the transesophageal echocardiography, which confirmed the presence of a pedunculated mass (19/5 mm), but attached only to the pacemaker lead, with multiple sites of binding, beginning from the right atrium (Figure 4a; Video 2, Supplementary File); no supplementary involvement of the tricuspid valve and myxomatous appearance of the posterior leaflet with hypermobility and rupture of chordae (Figure 4b), features that led to the false interpretation of tricuspid valve involvement in transthoracic images. 

**Figure 5:**
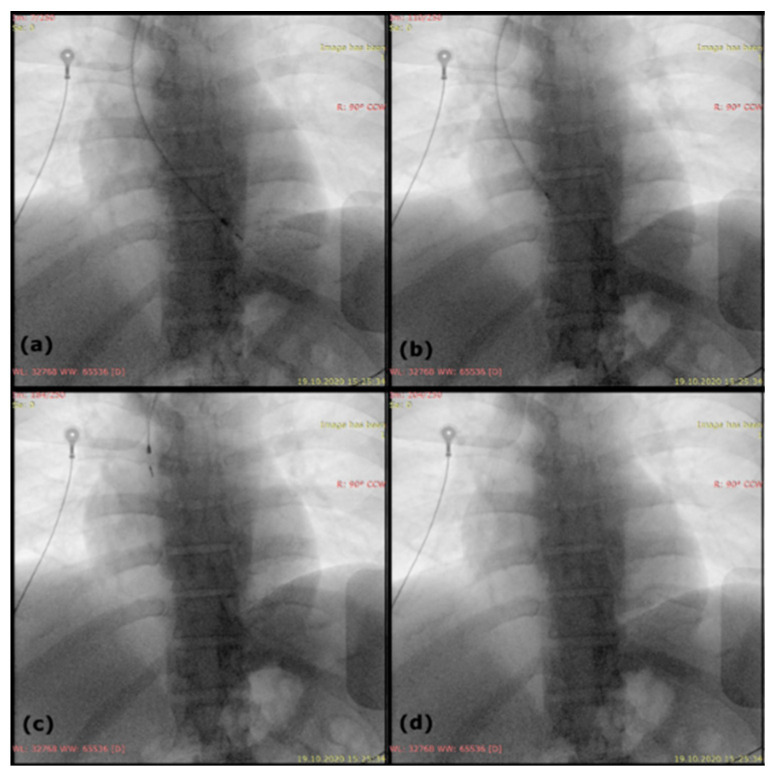
Antero-posterior fluoroscopic view of the lead extraction: (**a**) lead in the right ventricle; (**b**) lead in the right atrium; (**c**) lead in superior vena cava; (**d**) fluoroscopic view after lead extraction.

**Figure 6:**
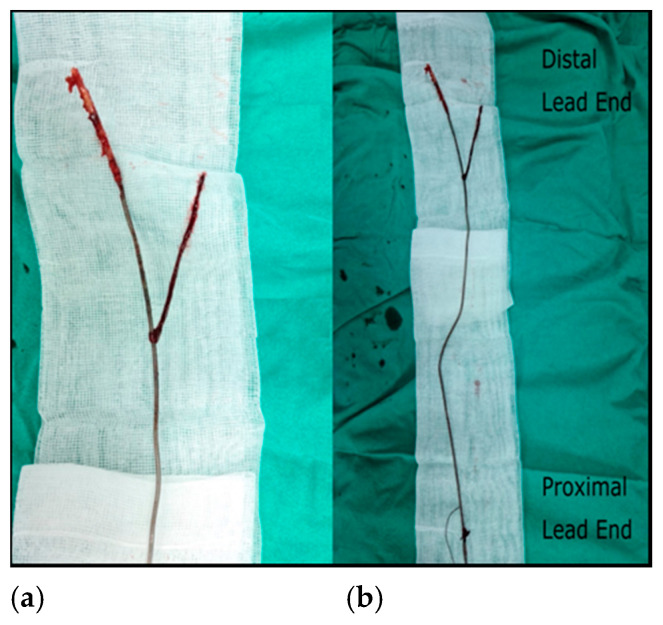
Extracted lead: (**a**,**b**) fibrotic capsule that encased the lead along the distal end and distal region of the lead and large pedunculate vegetation attached (approximately 38/6 mm).

After an increase in the hemoglobin value to 8.6 g/dl (with red blood cell transfusions), according to the current practice indications, we decided to remove the entire pacing system along with the vegetation. We used the superior venous approach since the lead was accessible via the implanted cephalic vein. We unscrewed the lead connector, removed the pacemaker generator and freed the lead from the surrounding conjunctive tissue in the pocket, then cut the proximal end of the lead together with the connector. A straight stylet of 58 cm in length was inserted in the remaining lead and secured with non-absorbable sutures. Subsequently, we removed the fibrous tissue that encompassed the lead with the help of special extraction tools—mechanical sheaths (Byrd Dilator Sheath Sets, Extra Long, Outer Sheath ID/OD 13.1/15.2 French and 14.1/16.3 French, Cook Medical). The set of two telescoping dilator sheaths was advanced over the lead under fluoroscopic monitoring and carefully manipulated to disrupt the scar tissue and break the fibrous adhesions from the binding sites (the costoclavicular region, the right ventricle—from the tricuspid valve to the right ventricular apex); because the lead had not been freed by the time the sheaths neared the myocardium, the outer sheath was advanced to the myocardium. Using continuous firm traction, while the sheath countered the traction and supported the myocardial wall, we were able to remove the unbroken ventricular lead. Fluoroscopic images during the procedure can be seen in Figure 5 and Video 3 (Supplementary File). The extracted right ventricular lead with fibrous tissue at the distal end of the lead and a much larger vegetation (38/6 mm) found with echocardiography can be seen in Figure 6. The lack of correlation between the estimated and the real dimension of the vegetation comes from the atypical, very elongated form of the vegetation initially attached to the lead. Infective material along the lead course generally does not cause typical vegetations of measurable size, and both transthoracic and transesophageal echocardiography may be falsely negative, because of the pedunculated, low echogenicity vegetation that moves out of phase with the structure to which it is attached. Excised tissue from the vegetation was submitted for histopathological and microbiological evaluation. No microorganisms were detected, and the histopathological features were typical of sterile vegetation. 

**Figure 7:**
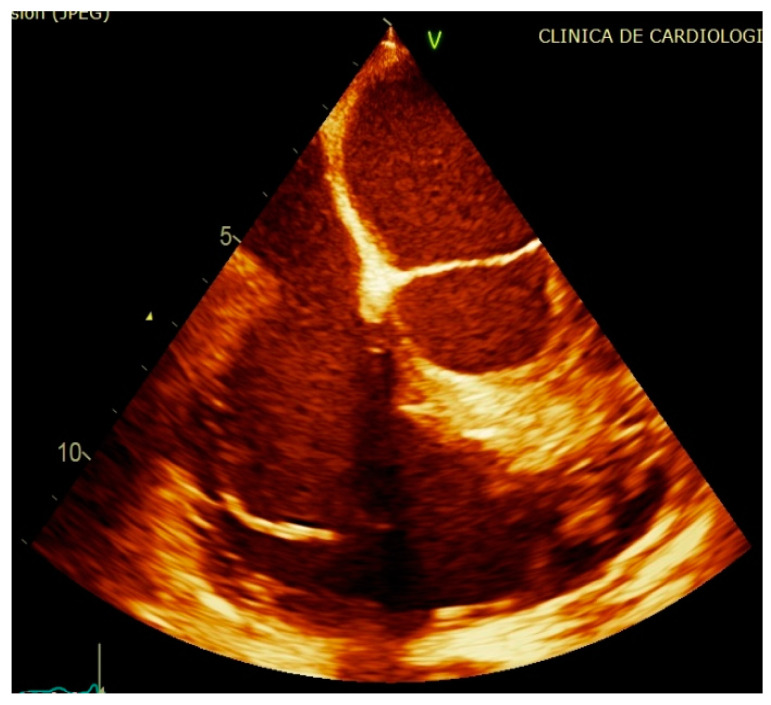
Transesophageal two-dimensional echocardiography: tricuspid chordae rupture, no residual vegetation, no pericardial effusion.

Postprocedural transesophageal echocardiography revealed mild tricuspid regurgitation secondary to posterior leaflet chordae rupture (Figure 7, Video 4, Supplementary File), no residual vegetation, no pericardial effusion.

**Figure 8:**
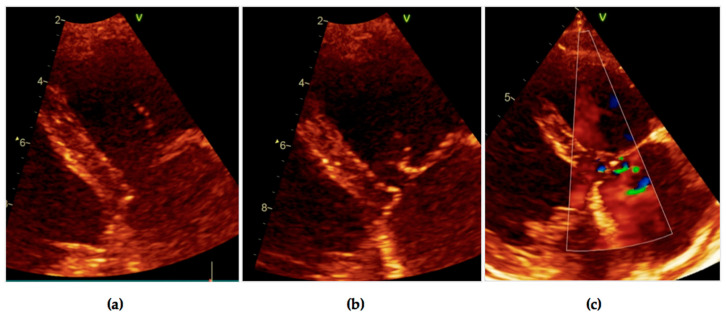
Transthoracic echocardiography, modified parasternal long axis–posterior leaflet of the tricuspid valve with chordal rupture, minor tricuspid regurgitation. (**a**,**b**) posterior leaflet of the tricuspid valve with chordal rupture; (**c**) minor tricuspid regurgitation.

The patient was discharged after 6 weeks of antibiotic therapy, with improved clinical status, persistent anemia, and elevated serum creatinine. A one-month follow-up revealed a favorable evolution, without the reappearance of symptomatology and further increases in hemoglobin value (10.4 mg/dL), no inflammatory syndrome, normal renal function, and minor tricuspid regurgitation due to chordae rupture; no additional cardiac masses (Figure 8; Videos 5 and 6, Supplementary File). Considering the electrocardiographic aspect and the repeated 24 h Holter monitoring examinations, both in normal ranges, corroborated the lack of syncope or presyncope in the last year, during which the patient did not benefit from pacing, so we decided to follow the patient, and postpone an implant, since no criteria for pacemaker implantation were met. 

This paper was written in accordance with the Declaration of Helsinki of 1975, which was revised in 2013. The patient gave verbal and written informed consent and fully authorized the authors to use his medical data for research purposes, as stated in the attached journal written informed consent and in the “patient informed consent” approved by the Hospital Ethics Committee (protocol according to the Order 1410/2016, issued by the Romanian Ministry of Health), both signed by the patient. 

## 3. Discussion

Late lead-related endocarditis is associated with higher mortality and morbidity than early-onset endocarditis and usually manifests with unspecific symptomatology, which leads to delayed diagnosis [9]. Although rare, this pathology should be suspected in patients with CIEDs and various signs or symptoms of infection that are unexplained or resistant to initial treatment [10]. The long periods of time between implant and lead endocarditis suggest a transient or persistent bacteremia as an ethological factor, since an inoculation at the time of implantation, as in the case of early-onset endocarditis, cannot be considered a cause anymore. Nevertheless, CIED patients are considered to be patients at low risk of developing bacterial endocarditis; according to the European infective endocarditis guidelines, which recommend antibiotic prophylaxis only in high-risk patients (patients with any type of prosthetic cardiac valve, with previous bacterial endocarditis, with any cyanotic congenital heart disease or with corrected congenital disease in the first 6 months if a prosthetic material was used, or lifelong if there is any residual shunt or regurgitation), patients with CIEDs should not receive antibiotic prophylaxis when performing invasive maneuvers [11]. Our patient, classified by the current guidelines in the category of negligible risk, did not receive antibiotic prophylaxis, a factor that probably led to bacteremia and vegetation formation, since no other possible entry sites were found and no history of other invasive procedures was present besides the two genitourinary tract procedures. 

Lead-related endocarditis is a pathology with difficult management, where complete system removal is required for successful treatment, associated with 4–6 weeks of antibiotics before new implantation; antibiotic therapy alone is not recommended, as it may increase the risk of recurrence and the 30-day mortality several fold [12], up to 31–66%, in contrast to 13–21% when early complete device removal is the chosen strategy [13]. Transvenous lead extraction is clearly the preferred method in patients with vegetations of 2 cm in diameter and smaller, with some authors considering percutaneous extraction even for larger vegetations because of the high level of open surgery-associated risks [14]. The involvement of cardiac valves or the need for a concomitant valve replacement or repair for infective endocarditis or significant retained hardware after percutaneous removal attempts give indication of surgery [15]. Transvenous extractions, instead, are not without risks; complications include myocardial avulsion, cardiac tamponade, vascular tear, hemothorax, and pneumothorax, with pulmonary embolism being the most frequent complication as a result of vegetation displacement during extraction [16]. Procedural mortality rates have been shown to be between 0.1% and 0.6% [17]. Procedural complexity may vary significantly according to lead type and features, but the most important factor is the time elapsed since the initial implant. 

The prevalence of endocarditis related to CIEDs varies widely among studies, from 0.5% to 2.2% of patients [18]. The incidence of infection associated with primary implantation is 2- to 5-fold lower than for revision procedures (primary 0.5–0.8%, revision 1–4%) over 1 to 3 years of the follow-up period [19,20,21]. Very late lead-related endocarditis is a rare entity, with sporadic mentions. After a literature search, we found only four lead-related endocarditis cases reported after more than 10 years since implantation [22,23,24,25], of which one patient had catheter dilation and stenting for superior vena cava syndrome (secondary to pacemaker lead fibrosis) performed four months before endocarditis diagnosis, a procedure that probably led to vegetation formation [24]. Two more cases of late lead endocarditis are described: the first, 7 years after initial implant [26] and the second, fungal endocarditis in an immunocompromised host, 9 years after implant [27]. All these cases have been addressed by surgery, with one exception: De Silva et al., who percutaneously removed the lead implanted 16 years earlier [23]. To our knowledge, the presented case would be the second case described in the literature with very late lead endocarditis and successful transvenous removal of the lead. 

Scar tissue encountered along the lead is usually the primary reason for partial or failed percutaneous removal of a lead. The duration of the device implantation appears to be an important factor in the formation of adhesions and, with it, problems with the extraction of leads. Most cardiovascular areas adjoining the leads can be affected by fibrosis. Scar tissue is present in multiple locations, with the venous entry site, subclavian area, and right ventricle as the most frequent sites, with the particularity that older leads tend to be more severely scarred, at the distal lead end [28]. Additionally, numerous case reports and studies have also described adhesions in the brachiocephalic vein [29], the arch into the superior vena cava, the superior vena cava itself [30], and the upper right atrium or tricuspid valve; occasionally, even papillary muscle and the chordae of the atrioventricular valve adhesions have been descried in autopsies [31,32]. In our patient, in which more than 12 years passed since the initial implantation, lead extraction posed a supplementary difficulty besides dwelling time, because it is associated with a large dimension vegetation; difficulties encountered were somehow typical to very late lead extractions, with the presence of extensive fibrosis at the venous entry site and in the right ventricle with neointimal fibrotic encapsulations of the distal end of the lead, which tend to occur in these patients over time. A passive fixation mechanism, as in the presented case, is usually associated with multiple adherences in the heart, because of the tines at the tip of the passive lead. Similar to passive lead fixation, dual coil ICD lead extractions are also difficult extraction procedures, as massive fibrous tissue growth along the lead is present, usually located in the innominate vein and superior vena cava [33]. Fixation mechanisms and electrode–tissue interactions have crucial implications for lead extraction. Active fixation leads have the advantage that once the screw is retracted, the tip can be easily detached from the endocardium, and the lead, being isodiametric, can be easily removed [34]. In the short term, the fibrous reaction and tissue ingrowth over the tines and into porous and grooved electrodes improves mechanical stability and intimate lead–tissue contact, but in the long term, this may also negatively influence the possibility of transvenous passive lead extraction. Encasement of the tines by fibrous reaction usually makes transvenous extraction more difficult than that of an active fixation isodiametric lead. The isodiametric or non-isodiametric design of the active or passive electrodes, respectively, also influence**s** the lead extractability: the presence of tines in passive fixation leads, which leads to the non-isodiametric design at the distal electrode, is the reason why scar tissue typically develops more frequently with this type of lead than with active fixation leads [35,36].

## 4. Conclusions

The number of patients who require CIEDs is significantly increasing every year and, as consequence, the number of CIED infections is on the rise worldwide. Late lead endocarditis is a pathology that is difficult to diagnose and therapeutic approaches involve multiple risks, considering the need for complete system removal; while very late lead endocarditis is a rarity, lead-related infective endocarditis is not an exception, and from our perspective, antibiotic prophylaxis in CIED patients should be reconsidered. 

## Data Availability

The data presented in this study are available on request from the corresponding author. The data are not publicly available due to privacy.

## Supplementary Materials

The following are available online at https://www.mdpi.com/2075-4418/11/1/96/s1, **Video 1.** Transthoracic two-dimensional echocardiography apical four-chamber view: large hypoechogenic hyper-pedunculated mobile mass at the level of tricuspid valve; **Video 2.** Transesophageal two-dimensional echocardiography: low echogenicity pedunculated mass attached to the pacemaker lead, with multiple sites of binding, with no supplementary involvement of the tricuspid valve and myxomatous appearance of the posterior leaflet with hypermobility and rupture of chordae; **Video 3.** Fluoroscopy during the lead extraction procedure, after freeing the lead from adhesions: traction of the lead from the right ventricular apex, through the tricuspid valve, right atrium, superior vena cava, and left subclavian vein, to complete lead removal; **Video 4.** Postprocedural transesophageal two-dimensional echocardiography: posterior leaflet chordae rupture, no residual vegetation, no pericardial effusion; **Video 5.** Transthoracic two-dimensional echocardiography parasternal short axis, no additional cardiac masses, posterior leaflet chordae rupture of the tricuspid valve; **Video 6.** Transthoracic two-dimensional echocardiography parasternal short axis, color Doppler: mild tricuspid regurgitation, with two small thin jets.

## References

[1] Rossi M., Musolino G., Serraino G.F., Renzulli A., Attila R. (2012). Device-Related Endocarditis and Infected Leads Extraction: The Dark Side of The Moon. Current Issues and Recent Advances in Pacemaker Therapy.

[2] Yuan S.-M. (2014). Right-sided infective endocarditis: Recent epidemiologic changes. Int. J. Clin. Exp. Med..

[3] Vilacosta I., Olmos C., De Agustin A., Lopez J., Islas F., Sarriá C., Ferrera C., Ferrera C., Sánchez-Enrique C., Vivas D. (2015). The diagnostic ability of echocardiography for infective endocarditis and its associated complications. Expert Rev. Cardiovasc. Ther..

[4] Cacoub P., Leprince P., Nataf P., Hausfater P., Dorent R., Wechsler B., Bors V., Pavie A., Piette J.C., Gandjbakhch I. (1998). Pacemaker infective endocarditis. Am. J. Cardiol..

[5] Klug D., Lacroix D., Savoye C., Goullard L., Grandmougin D., Hennequin J.L., Kacet S., Lekieffre J. (1997). Systemic infection related to endocarditis on pacemaker leads: Clinical presentation and management. Circulation.

[6] Singh N., Langer V., Chadha D.S., Ghosh A.K., Sengupta S., Gupta R., Dugal J.S. (2013). Percutaneous removal of transvenous pace-maker leads using an extraction device. Med. J. Armed Forces India.

[7] Ruttmann E., Hangler H.B., Kilo J., Höfer D., Müller L.C., Hintringer F., Müller S., Laufer G., Antretter H. (2006). Transvenous Pacemaker Lead Removal Is Safe and Effective Even in Large Vegetations: An Analysis of 53 Cases of Pacemaker Lead Endocarditis. Pacing Clin. Electrophysiol..

[8] Bongiorni M.G., Kennergren C., Butter C., Deharo J.C., Kutarski A., Rinaldi C.A., Romano S.L., Maggioni A.P., Andarala M., Auricchio A. (2017). The European Lead Extraction ConTRolled (ELECTRa) study: A European Heart Rhythm Association (EHRA) Registry of Transvenous Lead Extraction Outcomes. Eur. Hear. J..

[9] Osmonov S., Ozcan K.S. (2013). Cardiac device-related endocarditis: 31-Years’ experience. J. Cardiol..

[10] Scheffer M., van der Linder E., van Mechelen R. (2003). Pacemaker lead endocarditis. A rare diagnosis with a varied presentation. Neth. Heart J..

[11] Habib G., Lancellotti P., Antunes M.J., Bongiorni M.G., Casalta J.P., Del Zotti F., Dulgheru R., El Khoury G., Erba P.A., Iung B. (2015). 2015 ESC Guidelines for the management of infective endocarditis. Eur. Heart J..

[12] Rundström H., Kennergren C., Andersson R., Alestig K., Hogevik H. (2004). Pacemaker Endocarditis During 18 Years in Göteborg. Scand. J. Infect. Dis..

[13] Özcan C., Raunsø J., Lamberts M., Køber L., Lindhardt T.B., Bruun N.E., Laursen M., Torp-Pedersen C., Gislason G.H., Hansen M.L. (2018). Infective endocarditis and risk of death after cardiac implantable electronic device implantation: A nationwide cohort study. EP Europace.

[14] Cho H., Kim M., Uhm J.S., Pak H.N., Lee M.H., Joung B. (2014). Transvenous pacemaker lead removal in pacemaker lead endocarditis with large vegetations: A report of two cases. Korean Circ. J..

[15] del Río A., Anguera I., Miró J.M., Mont L., Fowler V.G., Azqueta M., Mestres C.A. (2003). Surgical treatment of pacemaker and defibrillator lead endocarditis: The impact of electrode lead extraction on outcome. Chest.

[16] Meier-Ewert H.K., Gray M.-E., John R.M. (2003). Endocardial pacemaker or defibrillator leads with infected vegetations: A single-center experience and consequences of transvenous extraction. Am. Heart J..

[17] Wilkoff B.L., Love C.J., Byrd C.L., Bongiorni M.G., Carrillo R.G., Crossley G.H., Epstein L.M., Friedman R.A., Kennergren C.E., Mitkowski P. (2009). Transvenous lead extraction: Heart Rhythm Society expert consensus on facilities, training, indications, and patient management: This document was endorsed by the American Heart Association (AHA). Heart Rhythm..

[18] Klug D., Balde M., Pavin D., Hidden-Lucet F., Clementy J., Sadoul N., Rey J.L., Lande G., Lazarus A., Victor J. (2007). Risk factors related to infections of implanted pacemakers and cardioverter-defibrillators: Results of a large prospective study. Circulation.

[19] Catanchin A., Murdock C.J., Athan E. (2007). Pacemaker Infections: A 10-Year Experience. Heart Lung Circ..

[20] Nery P.B., Fernandes R., Nair G.M., Sumner G.L., Ribas C.S., Menon S.M.D., Wang X., Krahn A.D., Morillo C.A., Connolly S.J. (2010). Device-Related Infection Among Patients With Pacemakers and Implantable Defibrillators: Incidence, Risk Factors, and Consequences. J. Cardiovasc. Electrophysiol..

[21] Sohail M.R., Uslan D.Z., Khan A.H., Friedman P.A., Hayes D.L., Wilson W.R., Steckelberg J.M., Jenkins S.M., Baddour L.M. (2008). Infective Endocarditis Complicating Permanent Pacemaker and Implantable Cardioverter-Defibrillator Infection. Mayo Clin. Proc..

[22] Ahn Y., Kim N.H., Shin D.H., Park O.Y., Kim W., Jeong M.H., Cho J.G., Park J.C., Kang J.C. (2004). Pacemaker Lead Endocarditis Caused by Achromobacter xylosoxidans. J. Korean Med. Sci..

[23] De Silva K., Fife A., Murgatroyd F., Gall N. (2009). Pacemaker endocarditis: An important clinical entity. BMJ Case Rep..

[24] Leong R., Gannon B.R., Childs T.J., Isotalo P.A., Abdollah H. (2006). Aspergillus fumigatus pacemaker lead endocarditis: A case re-port and review of the literature. Can. J. Cardiol..

[25] Takigawa M., Noda T., Kurita T., Okamura H., Suyama K., Shimizu W., Aihara N., Nakajima H., Kobayashi J., Kamakura S. (2007). Extremely Late Pacemaker-Infective Endocarditis due to Stenotrophomonas maltophilia. Cardiology.

[26] Cho M.-S., Kim S.-H., Nam G.-B., Choi K.-J., Kim Y.-H. (2011). Very late-onset lead-associated endocarditis. Can. J. Infect. Dis. Med. Microbiol..

[27] Tandon R., Bansal R., Verma A., Wander G.S., Mutti J. (2018). Late onset pacemaker lead fungal endocarditis in an immunocompetent host: A rare entity. IHJ Cardiovasc. Case Rep. (CVCR).

[28] Smith H.J., Fearnot N.E., Byrd C.L., Wilkoff B.L., Love C.J., Sellers T.D., Database F.T.U.L.E. (1994). Five-Years Experience with Intravascular Lead Extraction. Pacing Clin. Electrophysiol..

[29] Kataoka S., Shoda M., Saito S., Yagishita D., Yazaki K., Higuchi S., Kanai M., Ejima K., Hagiwara N. (2021). Transvenous lead extraction in a patient with polysplenia and inferior vena cava defect. J. Cardiol. Cases.

[30] Tesloianu N.-D., Ignat A.-M., Corduneanu D., Petris A.-O., Tudorancea I. (2017). A non-conventional approach to 10-year-delayed extraction of pacemaker leads associated with recurrent infective complications. Anatol. J. Cardiol..

[31] Keiler J., Schulze M., Sombetzki M., Heller T., Tischer T., Grabow N., Wree A., Bänsch D. (2017). Neointimal fibrotic lead encapsulation—Clinical challenges and demands for implantable cardiac electronic devices. J. Cardiol..

[32] Stokes K., Anderson J., McVenes R., McClay C. (1995). The encapsulation of polyurethane-insulated transvenous cardiac pacemaker leads. Cardiovasc. Pathol..

[33] Segreti L., Di Cori A., Soldati E., Zucchelli G., Viani S., Paperini L., De Lucia R., Coluccia G., Valsecchi S., Bongiorni M.G. (2014). Major predictors of fibrous adherences in transvenous implantable cardioverter-defibrillator lead extraction. Hear. Rhythm..

[34] Bongiorni M.G., Giannola G., Arena G., Soldati E., Bartoli C., Lapira F., Zucchelli G., Di Cori A. (2005). Pacing and implantable cardioverter-defibrillator transvenous lead extraction. Ital. Heart J..

[35] Candinas R., Duru F., Schneider J., Lüscher T.F., Stokes K. (1999). Postmortem Analysis of Encapsulation Around Long-Term Ventricular Endocardial Pacing Leads. Mayo Clin. Proc..

[36] Novak M., Dvorak P., Kamaryt P., Slana B., Lipoldova J. (2009). Autopsy and clinical context in deceased patients with implanted pacemakers and defibrillators: Intracardiac findings near their leads and electrodes. Europace.

